# Abdominal Spasm Induced Urinary Incontinence in a Patient With Cerebral Palsy: The Diagnostic Utility of Urodynamics in Neurological Disorder Management

**DOI:** 10.7759/cureus.16524

**Published:** 2021-07-20

**Authors:** Ranveer Vasdev, Elizabeth Schlessinger, Nissrine Nakib

**Affiliations:** 1 Urology, University of Minnesota Twin Cities, Minneapolis, USA

**Keywords:** urodynamics, neurologic disorders, cerebral palsy, neurogenic bladder, spasticity

## Abstract

The presentation of incontinence in a patient with complex neurological disorders can vary substantially and depend on the location and nature of neurological injuries. In this case report, a 53-year-old female with cerebral palsy presents with recurrent episodes of catheter discharge and incontinence due to presumed *bladder* spasms. However, urodynamics (UDS) study reveals the spasms to be *abdominal* in origin. This unique case illustrates the diagnostic utility of UDS and important considerations when evaluating patients with complex medical and neurological disorders.

## Introduction

In the field of Urology, patients with neurogenic bladder (NGB) represent an important and complex population. NGB symptoms can range from urinary retention to bothersome urinary frequency and largely depend on the location of neuronal injury. With neurological lesions between the pons and T10, patients can present with an overactive bladder. With lesions above the pons or below T10, patients can present with an underactive bladder. However, neurological diseases causing lesions throughout the spinal cord and brain (such as spina bifida, Parkinson's disease, multiple sclerosis, etc.) can cause a mixed or variable presentation of NGB [1].

Urodynamics (UDS) provides a valuable evaluation of lower urinary tract function to make informed clinical decisions based on underlying pathophysiology and/or evaluating treatment efficacy. For patients with suspected NGB, UDS can be considered as part of the initial evaluation and follow-up per American Urological Association (AUA)/Society of Urodynamics, Female Pelvic Medicine and Urogenital Reconstruction (SUFU) guidelines [2]. While this is not a strong recommendation, it can be particularly useful in patients with complex symptoms to guide treatment.

Here, we present a patient with cerebral palsy who had an unexpected cause of urinary incontinence.

## Case presentation

Our patient, Ms. G, is a 53-year-old female with a history of spastic quadriplegia cerebral palsy who was initially evaluated by our urology team for recurrent urinary tract infections (UTIs). At that time, Ms. G had an existing indwelling foley catheter, placed for elevated post-void residual volumes while hospitalized for lower extremity pain. Before hospitalization and catheter placement, she had persistent urinary incontinence. Ms. G’s other medical history is significant for bipolar disorder, complex regional pain syndrome, and type II diabetes.

Ms. G subsequently made frequent emergency department (ED) visits for sudden bladder “spasms” associated with a sudden loss of urine and expulsion of her catheter. During spasm episodes, the patient complained of severe abdominal pain and leakage around the catheter. Many conservative measures were taken to manage her symptoms including anticholinergics, sympathomimetics, and Belladonna/Opium suppositories. The foley balloon was also inflated to 30 mL to prevent the catheter from becoming dislodged. However, spasms persisted. 

Outpatient cystoscopy was performed for further evaluation. This revealed mild trabeculation, scant purulence, and cystitis cystica. Interestingly, Ms. G experienced symptoms of bladder spasms during cystoscopy but no bladder contractions were visualized. To further elucidate her persistent abdominal pain, a CT scan was performed and revealed diffuse bladder thickening (consistent with cystitis seen on cystoscopy) as well as a moderate stool burden. Ms. G continued to localize symptomatic spasms as either “abdominal” or “bladder.” The suspected etiology at this point was cystitis exacerbating her underlying neurological condition. However, the duration and morbidity of her symptoms prompted further evaluation with UDS.

**Figure 1:**
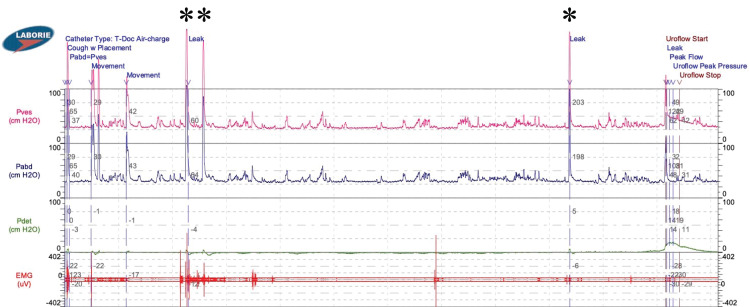
Abdominal spasms captured by urodynamics. Three large amplitude pressure signals due to abdominal spasms (*) were observed on vesical (Pves) and abdominal (Pabd) pressure tracings beginning after ~2 min of urodynamics. These signals coincided with the patient-reported spasm sensation and leakage in the absence of coughs.

Ms. G underwent UDS and during bladder filling, even at low volumes (50 mL), she experienced three symptomatic spasms, recorded as high amplitude signals on the abdominal pressure tracing, that all coincided with urinary leakage (Figure 1). Bladder capacity was found to be low (161 mL) with normal compliance. The remainder of the UDS test was unremarkable until the patient experienced an involuntary terminal detrusor contraction at a volume of 161 mL. There was no evidence of bladder outlet obstruction and her post-void residual was 0 mL. Given Ms. G’s history of cerebral palsy, her spasms were presumed to originate from the detrusor muscle, but UDS surprisingly suggested an abdominal origin.

Ms. G had well-established care with neurology for persistent quadriceps spasms. She was treated with baclofen, a (gamma-aminobutyric acid B) GABAB agonist, cyclobenzaprine, a central 5-HT2 receptor antagonist, and Botox injections into her quadriceps muscles without relief. Ultimately, she was started on dantrolene, an intramuscular ryanodine receptor antagonist, which initially relieved lower extremity spasms as well as abdominal spasms, and thereby her incontinence. Unfortunately, Ms. G did not tolerate dantrolene due to nausea and her symptomatic spasticity and incontinence returned after discontinuing the medication. However, once her UTIs were better controlled, she underwent a successful trial of void and was able to discontinue catheter use. As a result, her overall spasticity improved as did her incontinence.

## Discussion

We report an unusual case of stress urinary incontinence secondary to abdominal spasms in a 53-year-old woman with cerebral palsy. To our knowledge, this is the first reported instance of this clinical scenario. This case illustrates the challenges of evaluating urinary symptoms in patients with complex medical and neurological disorders as well as the utility of UDS to evaluate this patient population. 

In patients with cerebral palsy, the prevalence of symptomatic NGB can be as high as 30% [3]. However, the presentation of cerebral palsy NGB can vary substantially and include bladder spasms [4], diminished flow rates [5], and detrusor-sphincter dyssynergia [6]. The presentation of NGB is largely contingent on the location of the neurologic lesion(s). However, detrusor overactivity is the most common presentation of bladder dysfunction, affecting up to 59% of symptomatic children and adults with cerebral palsy [7]. In addition to detrusor overactivity, concurrent UTIs [8] or catheter irritation [9] can also provoke or exacerbate bladder spasms in patients with neurological dysfunction. Thus, the mechanisms underlying bladder spasms are multifactorial.

Before UDS, Ms. G’s symptoms were assumed to be bladder spasms, a common finding in patients with cerebral palsy [7]. However, symptoms did not improve with the standard therapy of anticholinergics and sympathomimetics [10]. Further workup with UDS revealed the cause of the incontinence to be stress-related as a consequence of abdominal spasms. These spasms were likely secondary to disinhibited hyperactive spinal reflex arcs seen in upper motor neuron lesions [11]. This discovery highlights the importance of keeping a broad differential when managing incontinence in patients with chronic neurological diseases.

The UDS correctly identified Ms. G’s spasms as abdominal, rather than the assumed bladder, emphasizing its utility in differentiating the etiology of incontinence [12]. UDS allows concurrent evaluation of bladder compliance, capacity, and contractility; however, as seen in this case, it can also identify extravesicular causes of incontinence such as abdominal spasms. Additional procedures, such as cystoscopy, may also be considered in the early evaluation of patients with neurological disorders [2].

This case illustrates the challenges of care in vulnerable patient populations. Managing life-changing illnesses such as bipolar disorder, chronic pain, and diabetes mellitus concurrently can complicate and potentially delay incontinence evaluation and management. Additionally, extensive medications used by this patient population may limit therapy options due to drug-drug interactions, side effects, or procedural contraindications. Incontinence management in patients with cognitive deficits can be especially challenging as care can depend on patient-reported urinary behaviors (ex. frequency, quality of void, etc.) [13]. As such, it is valuable to utilize a multidisciplinary, patient-centric approach to evaluate, diagnose and treat patients, like Ms. G, who have complex neurological conditions.

For patients with cerebral palsy or other neurological disorders, we recommend early UDS evaluation even if an empirical trial of anticholinergics is tried and is successful. If abdominal spasms are identified on UDS, treatment should follow the American Academy of Neurology guidelines for spasticity management in cerebral palsy patients and include trials of Botulinum toxin A injections into the abdominal muscles, diazepam, and/or tizanidine with careful consideration of side effects, comorbidities, and medication interactions [14].

## Conclusions

Stress urinary incontinence due to abdominal muscle spasms is a rare cause of bladder dysfunction in cerebral palsy patients. This case demonstrates that the UDS study is a valuable tool to investigate the etiology of lower urinary tract syndromes in patients with complex neurological dysfunction. Additionally, incontinence evaluation in this patient population should utilize a broad differential diagnosis and an interdisciplinary approach.
